# Transmission dynamics of COVID-19 in Algeria: The impact of physical distancing and face masks

**DOI:** 10.3934/publichealth.2020063

**Published:** 2020-10-30

**Authors:** Ali Moussaoui, El Hadi Zerga

**Affiliations:** Laboratoire d'Analyse Non linéaire et Mathématiques Appliquées, Department of Mathematics, University of Tlemcen, Algeria

**Keywords:** COVID-19, SIR compartmental model, basic reproduction number, mask protection, social distancing

## Abstract

We propose an SIR epidemic model taking into account prevention measures against coronavirus disease 2019 (COVID-19) such as wearing masks and respecting safety distances. We look for the conditions to avoid a second epidemic peak in the phase of release from confinement. We derive equations for the critical levels of mask efficiency, mask adoption (fraction of population wearing masks) and fraction of population engaging in physical distancing that lower the basic reproduction number *ℜ*_0_ to unity. Conclusions: For *ℜ*_0_ = 2.5, if at least 40% of people wear masks with efficiency 50%, and at least 20% of the population without masks (or anti-maskers) respect physical distancing measures, the effective reproduction number can be reduced to less than 1 and COVID-19 infections would plummet. The model predicts also that if at least half of the people respecting physical distancing, COVID-19 outbreaks with *ℜ*_0_ of about 3, would be theoretically extinguished without wearing masks. The results of this study provide an alternative explanation for the spread of the disease, and suggest some valuable policy recommendations about the control strategies applied to mitigate disease transmission.

## 1 Introduction

Late December 2019, The COVID-19 pandemic (coronavirus) began in Wuhan, China, and quickly spread around the world. Its novelty signifies that there is no existing community (herd) immunity and/or vaccine, which has required the great majority of the world's states to adopt strict measures such as lockdown or partial confinement. The impact of confinement measures on economic activity may become immense. Some sectors are totally paralysed: tourism, catering, while many employees could find themselves in short-time work [1].

**Figure 1.**
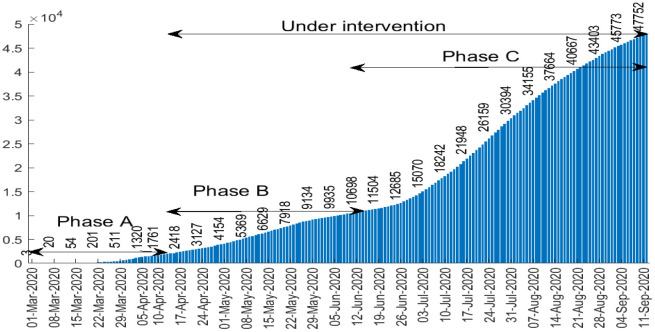
Cumulative number of confirmed COVID-19 cases in Algeria.

Algeria reported the first case of COVID-19 on 25 February 2020 [2],[3]. Since then, it has progressed rapidly and the number of cases tested positive grows exponentially each day. Figure 1 represents the cumulative number of detected cases of coronavirus in Algeria between March 01 and September 11, 2020 [2]. The cumulative number of COVID-19 cases is classified in three phases: The first phase (or phase A) corresponding to pre-intervention (e.g. from March 01 to April 10, 2020), the second phase (phase B) (between April 11 and June 13, 2020) corresponding to the intervention period when drastic measures were suddenly taken to slow the spread of the epidemic (closure of schools, restaurants, confinement, etc.). The third and final phase (phase C) (beginning from June 14, 2020), after curbing the epidemic, Algeria sees a rebound in COVID-19 cases. This came a few days after the government's decision to relax the measures adopted, particularly with the progressive reopening of stores, the resumption of public transport and other public places.

It may be difficult now to plan again strict confinement measures to slow spread of COVID-19 because it will affect the country's economy as all industries and import exports will cease. To deal with this situation, the population can undertake various non-pharmaceutical measures such as physical distancing and wearing masks [4]–[6].

Multiple epidemiological models have been proposed to predict the spread of COVID-19 epidemic and to evaluate the effectiveness of control measures to reduce and stop the virus spread, see for example [7]–[10]. In [11], the authors present a network model for the spread of COVID-19 pandemic based on the classical *SIR* model, random graph and percolation theory concepts to describe the impact of lockdown measures within a population.

The goal of this work is to propose a simple model allowing to examine the efficacy of near population-wide adoption of masks and physical distancing to bring down the basic reproduction number *ℜ*_0_, by reducing the number of people that are involved in physical interaction. *ℜ*_0_ is a conceptual parameter which provides some information about the expected speed through which a disease can propagate into a population. The organization of this paper is as follows. In the next section we present the *SIR* model without intervention and connecting its parameters to reported case data, we also derive the basic reproduction number and sharp estimates for the final size relation. In section 3, we present data in a way to get insight into the trend of the spread of COVID-19 during different phases of confinement in Algeria, we calculate the average rise of confirmed cases in each phase. In section 4, we examine the effects of physical distance and face masks on virus transmission by numerical simulation. We conclude the paper by section 5, where some detailed conclusions and discussions are presented.

## 2 The SIR model without intervention: (period until April 10, 2020)

To model the dynamic of the disease and predict the number of COVID-19 cases in Algeria, we use a standard SIR model described by Kermack and McKendrick [12]. The population is divided into three groups: susceptible, *S*, who can contract the disease, the infectives, *I*, those who have the disease and may transmit it, and the removed class, *R* where individuals out of the infection chain are put: isolated people, deceased and cured. The SIR model can be written as follows:

$$\frac{dS}{dt}=-\beta S\frac{I}{N}$$ (1)

$$\frac{dI}{dt}=\beta S\frac{I}{N}-\gamma I$$ (2)

$$\frac{dR}{dt}=\gamma I,$$ (3)

where *β* is the transmission rate per infectious individual and $\frac{1}{\gamma }$ is the infectious period. Recall that in the presented case of the simple SIR model, the basic reproduction number *ℜ*_0_ equals $\frac{\beta }{\gamma }$ [13],[14]. The basic reproduction number represents an indication of the initial transmissibility of the virus, i.e., the average number of secondary new infections generated by an infected person in an entirely susceptible population. In theory, when *ℜ*_0_ < 1, the epidemic cannot spread in the population. When *ℜ*_0_ > 1, the infected compartment *I* increases as long as ${ℜ}_{0}S>N=S+I+R$. Public health interventions can reduce the value of the basic reproduction number below one to prevent the epidemic.

### 2.1 Epidemic prediction

We now illustrate the predictions of the SIR model without intervention (1)–(3). The model is parameterized using the number of cases tested positive until 10 April (that is before intervention). We utilize the least squares method to estimate the parameters, which is implemented by the *fminsearch* command, found in the optimization toolbox in MATLAB. The aim is then to minimize the sum of the squared residual errors:

$$\hat{\theta }=argmin_{\theta \in \Theta }\sum_{t=1}^{n}{\left(R_{t}-R(t,\theta )\right)}^{2}$$

where *R_t_* is the cumulative number of reported COVID-19 and *R*(*t*,*θ*) is the theoretical confirmed cases calculated by the model on day *t*, *n* is the number of reported data. The set of parameter vectors *θ* constrained by a prespecified feasible region, here denoted by Θ.

**Figure 2.**
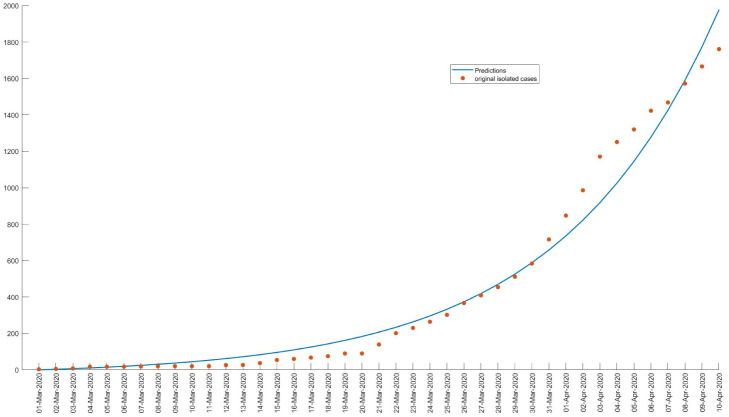
The curve corresponds to the expected observation given by the model, and the points correspond to the data.

The minimization leads to an estimate of *θ* = (*β*,*γ*). Based on the observed data the model parameters are estimated to *β* = 0.2044 and *γ* = 0.0971, thus *ℜ*_0_ = 2.1. The results are presented in Figure 2, we observe a good match with the data.

Let us return to the model (1)–(3). Let's determine what the final size of the epidemic will be in the absence of a complete intervention.

First of all, it is not difficult to prove that (see for example [15])

$$S(\infty )=\underset{t\to \infty }{lim}S(t)>0,\;I(\infty )=\underset{t\to \infty }{lim}I(t)=0,\;R(\infty )=\underset{t\to \infty }{lim}R(t)>0.$$

Equation (1) shows that

$$\frac{d}{dt}logS(t)=-\frac{\beta }{N}I(t).$$

Integrating between *t*=0 and *t* = ∞, we get:

$$logS(\infty )-logS(0)=-\frac{\beta }{N}\int_{0}^{\infty }I(t)dt.$$ (4)

Equation (2) can be rewritten as

$$\frac{dI}{dt}=-\frac{dS}{dt}-\gamma I.$$ (5)

Integrating between *t*=0 and *t* = ∞, we get:

$$-I_{0}=S_{0}-S(\infty )-\gamma \int_{0}^{\infty }I(t)dt.$$ (6)

Combining the two results (4), (6), and using the fact that S(∞)=N−R(∞) and *R*(0) = 0, we obtain

$$N-R(\infty )=S(0)exp(-{ℜ}_{0}\frac{R(\infty )}{N}),$$ (7)

If the entire population is initially susceptible, i.e., *S*(0) = *N* − 1 and *I*(0) = 1, the equation (7) can be written as

$$1-\frac{R(\infty )}{N}≃exp(-{ℜ}_{0}\frac{R(\infty )}{N}).$$ (8)

**Figure 3.**
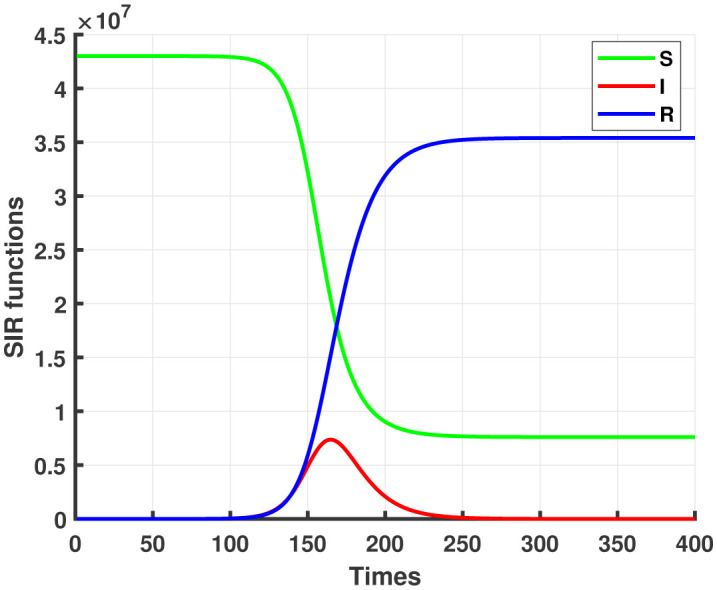
Solutions of SIR system (1)–(3), *S*_0_ = 43×10^6^ corresponds to the population size, $I_{0}=1,\beta =0.2044,\gamma =0.0971.$ Up to 82% of the population could likely contract the coronavirus if no control measures were imposed.

We find numerically $\frac{R(\infty )}{N}=82\%$. Figure 3 shows the simulation of infected cases with COVID-19 in a worst-case scenario, that is to say, without any intervention to modify the evolution of the epidemic.

## 3 Integration of intervention strategies

Like almost every country in the world, from March 23rd, but more rigorously from April 10th, Algeria has adopted drastic measures to limit the spread of the virus throughout our territory. On the basis of our observation, the pandemic management strategy in Algeria can be divided into three phases:

● **Phase A**: (from March 01 to April 10, 2020) corresponding to pre-confinement measures where some instructions are imposed as the prohibition of sporting, cultural and political gatherings, football matches were taking place without an audience.

● **Phase B**: (from April 11 to June 13, 2020) corresponding to lockdown of some cities and partial confinement of the others. This strategy has reduced contact and has surely reduced the spread of COVID-19 among the population, meaning gains for public health, while they reduced presence at the work-place, in restaurants, shops and other places of economic activity, involving economic costs, etc.

● **Phase C**: began from June 14 and corresponding to release from confinement, which is both progressive and flexible and in which they have decided the priority of activities according to their socio-economic impact and the risk of transmission of COVID-19.

**Figure 4.**
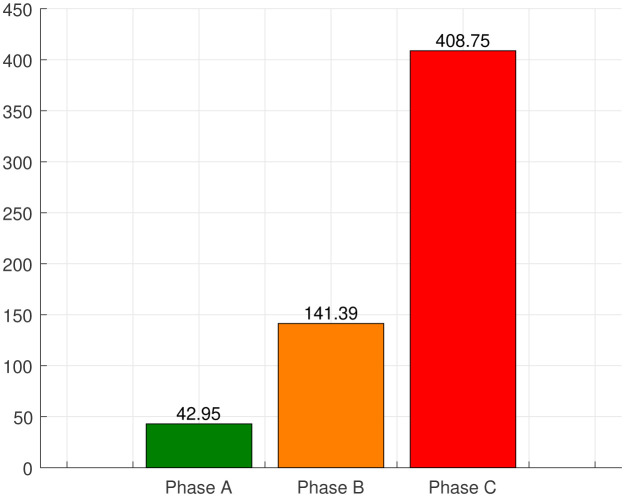
Average number of new cases in different phases.

Figure 4 gives the number of average newly reported cases in each phase. At the beginning of the epidemic, Algeria was not doing enough testing because there were few test kits available, making it difficult to assess the scale of the coronavirus epidemic in the country, which justifies the relatively slow spread of phase A. The actual number of infected patients in phase A must be higher as reported.

**Figure 5.**
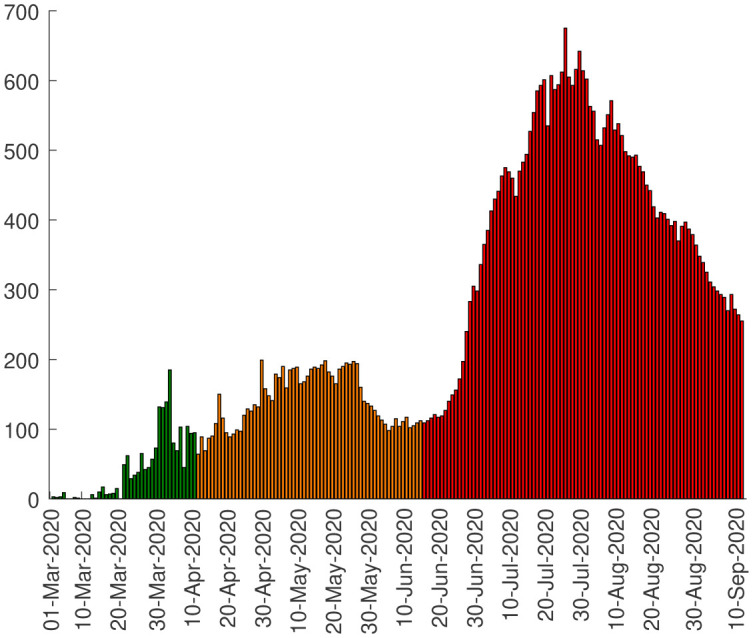
Daily cases in Algeria.

A few days after the outbreak began, the government has imported testing kits. In addition, the 20 laboratories spread across the country carry out over 2,500 COVID-19 tests per day on average against about 200/day at the beginning of the epidemic which justifies the significant increase in the number of daily cases of the new coronavirus in phase B. There is a rapid increase in confirmed COVID-19 patients in phase C as an effect of relaxation in confinement. We registered an average of around 43 new cases daily during phase A, 141 in phase B while 408 in phase C. On July 24, Algeria recorded 675 cases of coronavirus infections. This is the highest number ever since the first confirmed case in Algeria as shown in Figure 5. However, this number should be correlated with the increase in screening tests.

## 4 Face masks and physical distancing against COVID-19

The strict confinement measures taken in Phase B showed a slow and reasonably controlled spread of COVID-19, as intended. It was effectively achieving its goal of controlling the pandemic in the country, but on the other hand, it was causing some difficulties for daily betting and small businesses. Algeria, like several other countries, began process of release from confinement in order to minimize the economic damage caused by this health crisis.

A few days after relaxation of confinement, the number of new daily cases increased suddenly. If this situation is not well-handled, it could become uncontrollable, and it will be difficult for our hospitals to treat a large number of patients with coronavirus at the same time.

With the impossibility to return to strict confinement which could have even worse effects on the economy, face masks and physical distancing can reduce coronavirus transmission in communities where there are asymptomatic individuals or people with mild symptoms who are not diagnosed and continue to interact with other people.

In this section, we aim to investigate the effectiveness of physical distancing and face masks on the propagation of the virus. Let us assume that at the time of unconfinement, a proportion of the population “*m*” wears masks with some degree of protection *e* and a proportion “*d*” of the population practicing physical distancing without wearing masks. Based on our work [4], the SIR model with using mask and respecting social distancing is given by the system:

$$\frac{dS}{dt}=-\beta {\left(1-d\right)}^{2}{\left(1-em\right)}^{2}S\frac{I}{N}$$ (9)

$$\frac{dI}{dt}=\beta {\left(1-d\right)}^{2}{\left(1-em\right)}^{2}S\frac{I}{N}-\gamma I$$ (10)

$$\frac{dR}{dt}=\gamma I,$$ (11)

The effective reproduction number at the time of released from confinement is

$${ℜ}_{e}={ℜ}_{0}{\left(1-d\right)}^{2}{\left(1-em\right)}^{2}\frac{S(t)}{N}$$ (12)

and so long as the number of disease cases is small compared to the population size, this is approximately

$${ℜ}_{e}≃{ℜ}_{0}{\left(1-d\right)}^{2}{\left(1-em\right)}^{2}$$ (13)

One can easily confirm that in the simple case of free behavior, that this to say no mask (*m*=0) and without social distancing (*d*=0), *ℜ*_e_ = *ℜ*_0_. Similarly, in the case of 100% mask adoption level, (*m*=1) with guaranteed effectiveness (*e*=1), or when all individuals practice physical distancing (*d*=1), then *ℜ*_*e*_ = 0.

We now examine the tradeoff between the portion of population wearing masks with certain filter efficiency *e* and the rest of this population who practicing only physical distancing for which *ℜ*_*e*_ becomes equal to unity.

Equation (13) can be interpreted as a balance equation, saying that how the portion of population wearing masks depends on mask efficiency and on the fraction of population practicing physical distancing. To make some headway it may be useful to resort to a numerical representation of the situation arising from (13).

**Figure 6.**
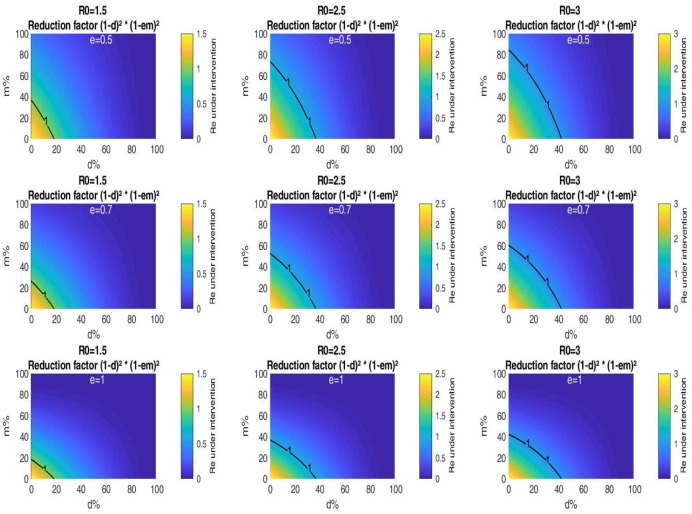
Impact of public mask wearing and physical distancing under different mask efficacy levels. The color indicates the resulting reproduction number from different initial values of *ℜ*_0_. The black line corresponds to *ℜ*_0_ = 1.

Figure 6 is a graphical representation of the critical fraction of the population respecting physical distance *d* versus the fraction of the population wearing masks *m* for various values of *ℜ*_0_ and *e*. As an example, assume that the mask efficiency is 0.5, and at least 30% of the population who don't wear masks engaging in physical distancing. In the case when *ℜ*_0_ = 3, Figure 6 shows that it is possible to lift totally confinement with success with masks worn by about 36% of the population. Whereas for *ℜ*_0_ = 2.5, we need on average 20% of the population wearing these masks. For *ℜ*_0_ = 1.5, we don't need to wear face masks to slow the spread of Covid-19 if 30% of the population practice social distancing. Figure 6 shows also that the higher the levels of mask efficiency, the more the level curve corresponding to *ℜ*_*e*_ = 1 is shifted at the bottom-left of each of the sub-figures, which means that there is a very close relationship between the portion wearing masks and the efficiency of these masks. As an example, for *ℜ*_0_ = 3, assume that 30% of the population practice physical distancing, then for a mask critical efficiency of 0.5, the mask adoption level is at least 36%. If the mask efficiency was 0.7, the mask adoption rate is reduced to about 25%. This rate is well below 18% if the mask efficiency was 1.

**Figure 7.**
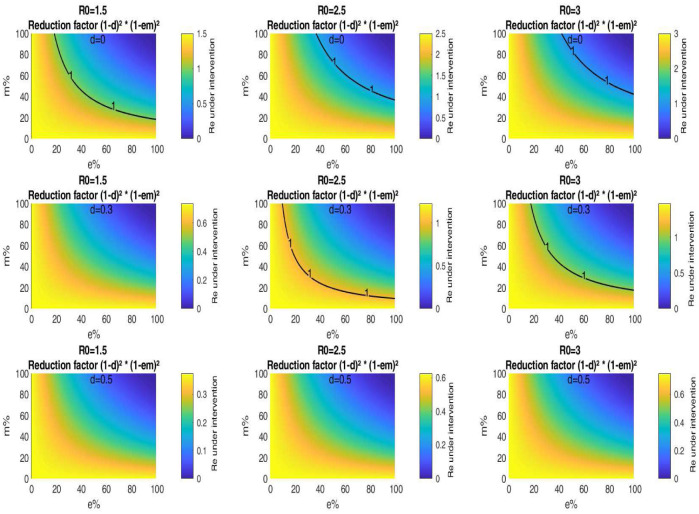
Impact of public mask wearing under the full range of mask adherence and efficacy scenarios for different values of *d*. The color indicates the resulting reproduction number from different initial values of *ℜ*_0_. The black line corresponds to *ℜ*_0_ = 1.

In Figure 7, we find the threshold of population wearing masks with some efficiency for which *ℜ*_*e*_ becomes equal to unity when a portion of the population engages in distancing measures. Figure 7 is a plot of the critical mask efficiency *e* versus the mask adoption level *m* for various values of *ℜ*_0_ and *d*. For *ℜ*_0_ = 3, if for example, 50% of the population that engages in physical distancing, then the effective reproduction number decreases with 75% of its original value according to this simplified model. Hence, the epidemic can be stopped without need face masks. Whereas when physical distancing can't be observed (*d*=0), it is needed to use masks worn by a portion *m* = 72% of the population and with efficacy almost equal to 60%. If 30% of the population respecting physical distancing and assume that the mask adoption level is 40%, then for *ℜ*_0_ = 3, Figure 7 indicates a mask critical efficiency of 50%. For *ℜ*_0_ = 2.5, the mask critical efficiency is reduced to about 30%. Whereas for *ℜ*_0_ = 1.5, the epidemic can be stopped without need face masks.

For a fixed *d*, Figure 7 shows that the higher the *ℜ*_0_, the more the level curve corresponding to *ℜ*_*e*_ is shifted at the top right of each of the sub-figures, which means that to avoid a second peak after unconfinement, it is necessary to ask a larger portion of the population to wear increasingly effective masks. Our model also indicates that if the half of the population respects physical distancing, it is not necessary to wear masks, unfortunately, this is not respected especially in closed places, which allows to encourage the general public to wear masks.

## 5 Discussion and conclusion

Equations were derived that give critical levels of portion of the population practicing physical distancing, mask adoption and mask efficiency that lower the effective reproduction number *ℜ*_*e*_ of a SIR-based epidemiological model to unity. These critical levels correspond to a strong reduction, towards zero, of the final number of cumulative infections. It would be much more effective for the population to adopt masks that exceed the critical mask efficiencies derived herein, and the people who don't wear masks must maintain physical distance.

To estimate the effective reproduction number, based on our simplified model, we thus needed an estimate of the following two parameters:

1. The portion of the population that engages in physical distancing.

2. The portion of the population that wear masks.

So, if face mask usage and physical distancing were to become a common behaviour, one can expect the number of diseases and deaths from COVID-19 to be reduced, especially if population compliance is high until such time as a pandemic vaccine becomes available.

Our research indicates also that public mask wearing is likely beneficial as source control when worn by persons shedding infectious coronavirus when physical distancing is not possible in public spaces. Finally in the last section we discuss the biological significance of our results.

Note that the model used here is highly simplified, it assume that the population has homogeneous mixing, with a fixed product of the number of contacts per day and of the probability of infection per contact. However, in some situations, for example at home, one would not normally expect people to wear masks, such limitations also apply to physical distancing. In the future, we plan to develop mathematical models taking into account the daily movements of individuals in different places, home, schools and universities, workplaces, transport and public places, shops where the risks of infection are different.
